# Effect of using a mobile drug management application on medication adherence and hospital readmission among elderly patients with polypharmacy: a randomized controlled trial

**DOI:** 10.1186/s12913-023-10177-4

**Published:** 2023-11-02

**Authors:** Hossein Poorcheraghi, Reza Negarandeh, Shahzad Pashaeypoor, Javad Jorian

**Affiliations:** 1 Dept. of Community Health and Geriatric Nursing, School of Nursing and Midwifery, Tehran University of Medical Sciences, Tehran, Iran; 2 Nursing and Midwifery Care Research Center, School of Nursing and Midwifery, Tehran University of Medical Sciences, Tehran, Iran; 3 Dept. of AI, Faculty of Engineering, Mashhad Branch, Islamic Azad University, Mashhad, Iran

**Keywords:** Elderly, Polypharmacy, Drug adherence, Application, Mobile phone

## Abstract

**Background:**

Adherence to complex drug regimens and polypharmacy are among the challenges of old age, which may negatively affect their motivation to continue drug therapy or lead to incorrect drug consumption. The present study was conducted to evaluate the effect of using a mobile drug management application on medication adherence and hospital readmission among polypharmacy older adults.

**Methods:**

In this randomized controlled trial study conducted in 2022, with Trial Registration Number (IRCT20191231045966N1) (18/07/2021), 192 Iranian older adults with polypharmacy were selected according to the inclusion criteria and allocated to case and control groups using the block randomization method. The data collection tools included a demographic questionnaire, case report form, and Morisky Medication Adherence Scale. The intervention was done using a mobile drug management application. Drug adherence was measured at baseline and both with hospital readmission were measured after 8 weeks. The collected data were entered into the SPSS software version 22 and analyzed using descriptive (frequency, percentage, mean, standard deviation) and inferential (Chi-square, Fisher’s exact test, independent t-test) statistics.

**Results:**

The case and control groups were homogeneous in terms of demographic variables and drug adherence level before the intervention. A significant difference was found in the drug adherence level after using the app (p < 0.001). Moreover, a significant difference was found in adverse events, including re-hospitalization due to disease aggravation, re-hospitalization due to error in medication consumption, falling, hypo or hypertension, and hypo or hyperglycemia, and medication use accuracy between the groups after the intervention (p < 0.05).

**Conclusion:**

The results showed that using a mobile drug management application that meets the specifications of older adults can improve drug adherence, reduce the adverse events and pave the way for a better disease period management.

**Supplementary Information:**

The online version contains supplementary material available at 10.1186/s12913-023-10177-4.

## Background

Population ageing has turned into one of the most important public health challenges in recent years, a phenomenon that has affected Iran at a higher speed compared to other countries [1]. Ageing is associated with changes in different body organs. In this period of life, chronic diseases threaten the elderly person’s health so that the elderly population is the largest drug consumer in different societies [2]. Under these circumstances, the elderly should take complex drug regimens for their treatment process leading to a phenomenon known as polypharmacy. There are different definitions for polypharmacy; however, it most commonly refers to daily using five medications or more [3]. Polypharmacy is extensively prevalent in different societies, for example, its prevalence is about 36% in England where older adults above 65 years constitute one-fifth of the population [4]. The mean prevalence of polypharmacy is 23.1% in Iranian older adults [32.7% in women and 15.2% in men) [5]. This phenomenon is associated with adverse consequences, including; drug interactions, error in medication consumption, increased side effects, re-hospitalization, falling, functional and cognitive disorders, imposing financial burdens on the health care system, and finally disability and death. Polypharmacy has a direct relationship with reduced physical activity, motion disorder, decreased appetite, and depression in the elderly, and may seriously affect their medication adherence and quality of life [6].

Medication adherence is one of the challenges associated with polypharmacy in the elderly population. Medication adherence occurs when a patient takes their medications according to the prescribed dosage, time, frequency, and direction [7]. Effective medication adherence reduces treatment costs, accelerates the recovery process, stops disease progression, and prevents re-hospitalization [7]. The personal factors related to drug non-adherence are divided to two categories of intentional non-adherence including self-drug discontinuance and unintentional non-adherence including problems such as forgetfulness, visual impairment, and inability to move, among which forgetting is a very important cause of poor medication adherence [8]. Poor medication adherence has been reported in 26–59% in older adults, depending on the population and methods used to assess drug adherence [9]. Also a study on 24,000 Iranian elderly patients showed that 62% of them forgot to take their medications [10]. Poor drug adherence is associated with worsening of the elderly patients’ health condition, increased hospitalization period, and risk of disease progression, disability, and death [11].

Technology advances have opened new horizons for management of chronic diseases and improvement of health care services. Use of mobile phone facilitates monitoring of treatment process and health care providers-clients’ relationship [12]. Today, mobile health (mHealth) is one of the most up-to-date types of health care interventions that can play an effective role in promoting older adults’ health. Mobile health apps have features such as; messaging, alarming, and event reminder. These features can be used to overcome problems such as forgetfulness, so it’s one of the most popular method among technology-based strategies for drug use management [13].

However, many apps that are designed for the general population are not customized for using by the elderly, which comprise a large population with various health needs so little interaction is observed between them and these apps [14]. Most of older adults suffer from impaired vision and hearing and have tough problems for using smartphones, which are usually ignored in designing these apps [15]. Moreover, the apps designed for older adults are usually in languages other than Farsi, rendering them practically useless for Iranian older adults. Persian drug reminder apps only remind medication use time and have failed to meet elderly needs. Ease of use, interesting user interface, font adjustment capability, and use of appropriate warm colors to compensate any vision impairment are among the factors that should be considered in designing a suitable app for this age group. In other words, a drug management app should be designed in such a way that even those who are only able to read and write, can use it [16].

In this regard, the Medisafe, a reminder alarms app, was designed to monitor the older adults’ blood pressure and boost drug adherence level. The results showed that medication adherence improved and blood pressure preserved in the normal range in patients in intervention group. However, despite improving medication adherence among the elderly, the mobile application did not provide features such as information about the drug use instructors [17]. As another case, the AlerHTA app, an alarm reminder, aiming to increase drug adherence, drug literacy promotion and aiming reminding time of drug use for patients with hypertetion. The results showed higher medication adherence in the elderly patients in the intervention group. Considering medication adherence promotion in studied elderly, this application was not specifically for older adults and only served as a medication reminder [14]. Additionally, in some cases, the designed apps failed to achieve their objectives completely. For example, in one study, no significant difference was observed in blood pressure control between elderly patients in case and control groups after using an educational app [18].

Since the available apps do not meet the older adults’ needs for medication use management due to their special conditions, they need an app that has the highest congruence with their physical and mental conditions. A review of the literature suggests that the older adults needed items are not considered in the available apps. Therefore, it was decided to design an app that has the highest congruence with their needs. Furthermore, there are controversies data regarding the effectiveness of drug management apps in promoting drug adherence and its associated consequences. Insights into the potential benefits of an expert-designed mobile application in promoting drug adherence among older adults; this controlled trial study was conducted to evaluate the effect of using a mobile drug management application on medication adherence and hospital readmission among polypharmacy older adults.

## Methods

### Study design, sampling and data collection

A randomized controlled trial study was conducted in April-June 2022. The research population consisted of older adults presenting to a hospital in Tehran, Iran. This hospital is a prominent health center for the geriatric population and has geriatric-specific clinics and services specializing in both acute and chronic diseases. This hospital welcomes clients for routine and periodic health checkups based on their health needs and physician’s order in all days of week.

**Fig. 1:**
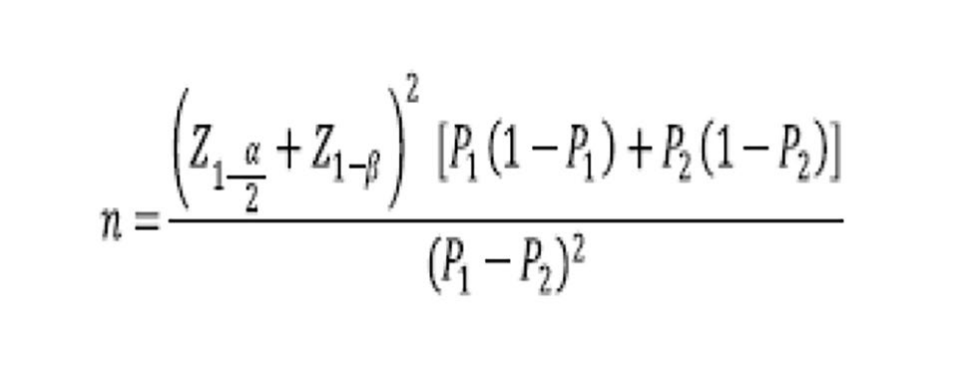
Sample size calculation formula

The inclusion criteria were age above 60 years, daily intake of more than 5 types of drugs, ability to read and write based on being able to fill out necessary forms in clinic, ability to communicate with, having smartphone ownership based on their self-report and a positive history of cardiovascular disease, diabetes, hypertension, or COPD. The exclusion criteria were a history of cognitive diseases, use of expensive and hard-to-find drugs, a positive history of special hard-to-treat diseases like cancer, use of injection drugs (e.g., insulin-dependent diabetic patients were excluded), not receiving clinic follow-up, unwillingness to participate in the study, and death. To calculate the sample size, for an intervention that could reduce poor adherence by 20% with a confidence interval of 95% and power of 80%, 86 participants were needed in each group [10]. Considering a loss to follow-up of 10%, 96 participants were needed for each group (Fig. 1) [19].

Recruitment process occurred among inpatient and outpatient clients referring to hospital. The researcher was present in hospital reception to assess the eligible elderly based on inclusion and exclusion criteria checklist. Among these, two hundred and fourteen older adults who were compatible with inclusion and exclusion criteria were selected with simple random method. Finally, 192 older adults volunteer to participate in this study were assigned to case and control groups using block randomization method with a block size of four.

The allocation sequence was generated using www.randomization.com [20]. An opaque envelope was used for allocation concealment. At the time of enrollment, according to the order by which the participants entered the study, one of the envelopes was opened in order and the allocation group was determined. The primary outcome was medication adherence and the secondary outcome was the adverse events experienced during the study. Medication adherence was measured at baseline and eight weeks after the intervention. The data collection tools were a demographic questionnaire, a researcher-made adverse events questionnaire, and the Morisky Medication Adherence Scale. The demographic questionnaire was used to collect data on age, sex, marital status, education level, income sufficiency, type of disease, list and number of chronic medications used by patients for confirming polypharmacy, method of information acquisition about the consumed drug, and the most used feature in mobile phone. The Morisky Medication Adherence Scale was developed to evaluate medication adherence by Morisky et al. in 2008 [21]. This scale was translated to Persian according to the Iranian culture and validated by Kooshyar et al. [22]. This scale contains eight questions. Response categories are yes/no for the first seven items and a 5-point Likert response from never to always for the last item. A score < 6 indicates low adherence, a score of 6 to < 8 shows moderate adherence, and a score of 8 represents high adherence [21]. In addition, pill count method was also used to assess medication adherence. Pill count method was taken for each intervention group participant. The control group only received medication for their prescriptions. In first visit and after having the application set for older adults in intervention group, the investigator confirmed patient enough pills supply until next visit. In second clinic visit, information was obtained on the number of pills returned and dispensed. The difference between the number of pills received at the previous visit and the number of pills returned represented the number of pills assumed used by pill count. This number was compared with the number of days that had elapsed between the previous visit and the current visit. Ratio ranges between 0 and 1 where the maximum value is 1. Medication correct use was defined as having value ≥ 0/85 − 1 [23, 24]. A researcher-made adverse events questionnaire was used to measure the occurrence five complications; re-hospitalization due to disease recurrence, re-hospitalization due to error in medication consumption; determined by doctor, falling from the bed at home by patient report, hypo or hypertension and hypo or hyperglycemia measured by the patient at home or health care providers in hospitals in periodic visits. Accuracy and reliably of patient response to this case report form, was examined by their self-declaration and medical records.

This form was developed for this study and in order to assess the face and content validity of this case report form, a panel of six geriatric experts was asked to rate the relevancy, clarity, simplicity, and necessity of each question using Likert scale. All members of the panel had relevant knowledge in either usability evaluations or elderly needs. Subsequently, the Content Validity Ratio (CVR) and Content Validity Index (CVI) were calculated according to previous studies. Content validity was considered to be acceptable when CVI and CVR were at least 0.78. In order to confirm the reliability of the questionnaire, two methods of internal reliability and test-retest reliability were used. In this sense, Cronbach’s alpha was calculated as the measure of internal reliability. Cronbach’s alpha equal to and above 0.7 was considered as the minimum acceptable value. To measure the test-retest, a total of 20 older adults was asked to score the questionnaire twice with a two-week interval. Then, the Pearson correlation coefficient was calculated between the two sets of scores.

### Intervention

In order to design the application used in this study, an in-depth review was conducted on the existing medication reminder application (both in Farsi and English) to identify their week points in order to solve these issues in new design. A drug management app compatible with android operating system with features such as ease of use, adjustable font and text size, use of proper colors in background and app item, saying the name of the drug and showing its picture while playing a reminder for its use, and using phrases like “Dear mother/father! It’s time for your drug” was designed for Iranian older adults in Persian language for the first time. The drugs name and picture was recorded when setting the reminding alarm. All educational content in this app were reviewed and confirmed by three geriatric experts. In order to protect the privacy and security of app users, the most up-to-date programing codes were used to design the app. As a pilot test and finding the possible flaws and ensuring its correct function on mobile phones, it was installed on the mobile phones of 10 elders. They were asked to report any problem and their ideas about app improvement and its ease of use. The comments of geriatric experts in this field and older adults were both used in design phase. After receiving the final correct operability confirmation and obtaining informed consent, the app was installed on the mobile phones of the participants in the intervention group. Face-to-face training on how to use the app was offered to each participant in a 60-minute session and medication use alarms were set. During the intervention, app users were contacted via phone calls or in routine hospital referrals to ask and check if there is any problem using the app to ensure to ensure intervention fidelity. Also the participants could contact the researcher through the phone number given to them to ask their questions. Number and therapeutic category of drugs were similar in the control and intervention groups. The participants in the control group received the routine care of the health center including periodical visits to evaluate the treatment process and required care. Considering the Covid-19 pandemic in Iran and the sensitive conditions of older adults, it was difficult to make the necessary arrangements for participation of the elders in the introduction session, which included various topic such as the objective of the study, using the app, and completing the questionnaire. To address this problem, the participants were grouped into different groups, and Covid-19 related protocols including social distancing, face mask use, and disinfection were strictly considered.

### Data analysis

Data were entered into the SPSS software version 22 and analyzed according to per protocol using descriptive (frequency for number of chronic medications, mean for age, percentage and standard deviation for related categories) and inferential (Chi-square for sex, education level, income sufficiency, type of disease, adherence level before and after intervention, Fisher’s exact test for marital status method of information acquisition about the consumed drug and the most used feature in mobile phone and finally independent t-test for age) statistics. In inferential analysis, demographic data of control and intervention group and adherence level before and after intervention in both group were compared with each other at baseline and 8 weeks. The effect size was calculated using the Cramer’s statistic. P values less than 0.05 were considered significant.

**Fig. 2:**
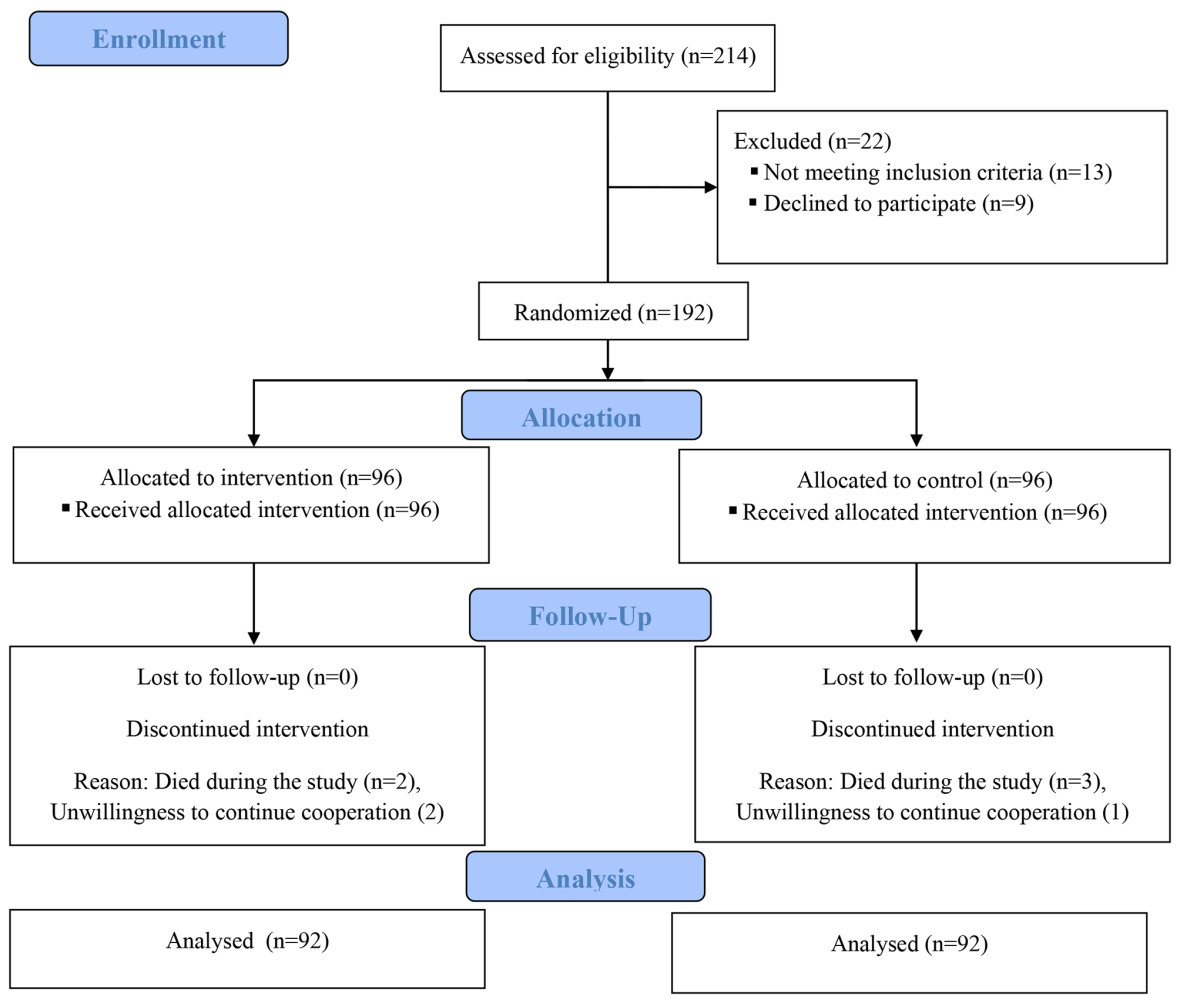
The flow of participants in the study (CONSORT Flow Diagram)

## Results

In the present study, 214 patients were evaluated according to the inclusion criteria, of whom 13 were excluded due to not meeting the inclusion criteria and 9 due to unwillingness to participate in the study. Then, the remaining 192 patients were randomly assigned to intervention and control groups. During the intervention, 4 patients were excluded from the control group (3 due to death and 1 due to unwillingness to continue the study because of travelling); moreover, 4 patients were also excluded from the intervention group (2 due to death and 2 due to unwillingness to continue the study; one for not clear reason, the other one for having his phone broken). Finally, data of 92 cases and 92 controls were analyzed (Fig. 2).

Demographic findings showed that the participants mean age was 69 ± 5.6 and 68.9 ± 5.2 years in the control and intervention group respectively, indicating no significant difference (p = 0.926). Mean total number of medications taken by control group was 6.53 ± 2.3 and 6.67 ± 2.4 at baseline and in follow-up respectively which indicate no significant difference P = 0.839. Also mean total number of medications taken by intervention group was 6.45 ± 2.1 and 6.55 ± 2.3 at baseline and in follow-up respectively which indicate no significant difference P = 0.874. Moreover, the two groups were homogenous in terms of sex, marital status, education level, income sufficiency, cardiovascular disease, hypertension, diabetes, COPD, method of information acquisition about the consumed drug, and the most used feature in mobile phone (p > 0.05) (Tables 1 and 2).

**Table 1:** Demographic information of the studied units in two groups

Variable		Control group (n = 96) N (%)	Intervention group (n = 96) N (%)	P-value
Sex^b^	Female	43(44.8)	47(48.96)	P = 0.665
	Male	53(55.2)	49(51.04)	
Marital status^c^	Single	3(3.13)	5(5.2)	P = 0.194
	Married	77(80.2)	74(77.1)	
	Divorced	3(3.13)	9(9.37)	
	widow	13(13.54)	8(8.33)	
Education level^b^	Primary school	11(11.46)	7(7.2)	P = 0.483
	Junior school	16(16.67)	15(15.6)	
	High school	27(28.12)	27(28.1)	
	Diploma	29(30.21)	25(26.2)	
	Academic education	13(13.54)	22(22.9)	
Income sufficiency^b^	Independent	85(88.54)	83(86.46)	P = 0.828
	Dependent	11(11.46)	13(13.54)	
Information acquisition method^c^	Physician and health workers	56(58.34)	54(56.25)	P = 0.776
	Family and friends	17(17.7)	8(8.33)	
	Books and pamphlets	8(8.34)	22(22.91)	
	Other	15(15.62)	12(12.51)	
The most used feature in mobile phone^c^	Message and calling	45(46.87)	41(42.7)	P = 0.756
	Radio	10(10.42)	9(9.38)	
	Applications	26(27.09)	28(29.17)	
	Camera	4(4.16)	2(2.08)	
	Internet	11(11.46)	16(16.67)	

* Chi-square test^b^, Fisher’s exact test^c^

**Table 2:** Disease status in each of the participants in the two groups

Type of disease		Control group (n = 96) N (%)	Intervention group (n = 96) N (%)	P-value
Cardiovascular disease^b^	Yes	51(53.12)	54(56.25)	P = 0.772
	No	45(46.88)	42(43.75)	
Hypertetion^b^	Yes	62(64.58)	64(66.67)	P = 0.879
	No	34(35.42)	32(33.33)	
Diabetes^b^	Yes	55(57.29)	47(48.96)	P = 0.311
	No	41(42.71)	49(51.04)	
COPD^b^	Yes	38(39.58)	47(48.96)	P = 0.766
	No	58(60.42)	49(51.04)	

* Chi-square test^b^

Evaluation of drug adherence level showed no significant differences between two groups before the intervention (p = 0.919). However, after the intervention, the difference was significant in the intervention group, and the participants with high adherence increased from 12.5 to 44.56%, indicating a significant difference (p < 0.001). According to the Cramer’s statistic, the effect size of the intervention on drug adherence was above moderate [25] (Table 3).

**Table 3:** Comparison of drug adherence levels between two intervention and control groups before and after the intervention

	Adherence level	Control group (n = 96) N (%)	Intervention group (n = 96) N (%)	P-value	Effect size^b^
Before intervention^a^	Low	47(48.96)	50(52.08)	0.919	
	Moderate	37(38.54)	34(35.42)		
	High	12(12.5)			
		(n = 92) N (%)	(n = 92) N (%)		
After intervention^a^	Low	40(43.48)	22(23.92)	< 0.001	0.315
	Moderate	37(40.22)	29(31.52)		
	High	15(16.3)	41(44.56)		

* Chi-square test^a^, Kramer’s statistic^b^

Comparison of the adverse events between the two groups showed a significant difference in re-hospitalization due to disease aggravation and error in medication consumption, falling, hypo or hypertension, hypo or hyperglycemia, and drug use accuracy according to the prescriber’s order based on the pill count method between the two groups (p < 0.05). Drug use accuracy ranges between 0 and 1 where the maximum value is 1. Medication correct use was defined as having value ≥ 0/85 − 1 (Table 4).

**Table 4:** Comparison of adverse events and drug use accuracy between control and intervention groups

Adverse events		Control group (n = 92) N (%)	Intervention group (n = 92) N (%)	P-value
Re-hospitalization due to disease aggravation^a^	Yes	43(46.74)	17(18.48)	< 0.001
	No	49(53.26)		
Re-hospitalization due to error in medication consumption^a^	Yes	22(23.91)	9(9.78)	P = 0.017
	No	70(76.09)	83(90.22)	
Falling^a^	Yes	21(22.82)	7(7.61)	P = 0.007
	No	71(77.18)	85(92.39)	
Hypo or hypertension^a^	Yes	33(34.78)	14(15.22)	P = 0.004
	No	60(65.22)	78(84.78)	
Hypo or hyperglycemia^a^	Yes	22(25)	7(7.61)	P = 0.011
	No	69(75)	85(92.39)	
Drug use accuracy^a^	≥ 0/85 − 1	33(35.87)	65(70.65)	< 0.001
	< 85	59(64.13)		

* Chi-square test^a^

## Discussion

The present study was conducted to evaluate the effect of using a drug management app on drug adherence and adverse events in polypharmacy adults. The results showed that using an app customized for the special conditions of the older adults improves medication adherence. In line with results of present study, Najafi et al. conducted a study to investigate the effect of using a mobile phone-based application on medication adherence in patients with heart failure during three months. The results showed a significant increase in the medication adherence score after using the app. Medication adherence changes were more significant in the intervention group compared to the control group, indicating improved medication adherence in people with heart failure after using the app [26]. The results of the above study were consistent with the results of the present study, suggesting the positive effect of drug management apps on improved medication adherence. Santo K conducted a study to determine the effect of medication reminder applications on drug adherence in patients with coronary heart disease in three months. The primary outcome was the medication adherence level and the secondary outcomes were the blood pressure and cholesterol level control. Mean score of medication adherence was significantly higher in app users compared to control group, which is similar to the present study [27]. In another study, Li et al. evaluated the effect of a smartphone application on medication adherence in 24 polypharmacy patients with a mean age of 59.5 years for one year in Australia. In this study, app users in intervention group received medication regimen and educational messages through the app. Participants were required to report each time they took a medication via the app. The control group received routine care including routine visits. Both groups were evaluated three times, including one, three, and twelve months after the intervention. The results showed a 4.37 times higher improvement in the medication adherence in the intervention group compared to the control group in the third assessment [28]. Although Li et al. did not exclusively focus on older adults and the participants age ranges 18–75 years, its results were consistent with the results of the present study, indicating improved medication adherence in drug management app users.

Baghei et al. studied the effect of a mobile educational application on medication adherence in hypertensive older adults. Comparison mean scores of medication adherence components showed significant difference in commitment to treatment and hesitation in implementation of treatment between two groups while no significant difference was observed in the blood pressure status between the intervention and control groups. However, in present study, blood pressure alterations reduced significantly in the intervention group compared to control group; therefore, the results are not consistent. This difference may be due to the follow-up duration. However, it can be concluded that mobile app education can improve medication adherence in older adult app users [18].

Habib et al. conducted a study to improve medication adherence following hospital discharge using a mobile application in Canada. This study was conducted on 49 patients with a mean age of 64.6 years assigned to two groups. The patients were followed for 30 days’ post-discharge. During the follow-up period, they were evaluated for medication adherence and re-hospitalization. At the time of discharge, the app was installed on the mobile phones of the patients in the intervention group. Drug management app named SAM, an alarm reminder app, with mission to improve medication adherence in older adults. According to the results, the medication adherence rate was 83.7% in the intervention group and 77.8% in the control group, indicating no significant difference [29]. Although it seems that the results of the above study and our study are not consistent, the reason may be the short follow-up time of this study. Moreover, in this study, only 65.2% of the patients in the intervention group used the app, which could be due to reasons such as failure to design an appropriate app for this group and lack of proper training for its use or not being user friendly.

The results of the present study showed that using the drug management app reduced adverse events including re-hospitalization due to disease aggravation and error in medication consumption, falling, hypo or hypertension, hypo or hyperglycemia, and promote medication use accuracy according to the pill count method. In this regard, Park et al. conducted a study to determine the effect of using digital health monitoring on readmission reduction in patients with health failure in the United States. This study was carried out on 58 patients and readmission rate was measured during 30 days. The results showed that overall 30-day readmission rate was 10% in these patients, while the mean readmission rate was 25% across the country indicating a significant reduction [30], which was consistent with results of present study. Findings suggest use of new technologies in health care system to have a significant reduction in the re-hospitalization rate. Furthermore, Sartori et al. conducted a study to evaluate the effect of educational intervention using the WhatsApp platform on medication adherence in hypertensive and diabetic patients. Intervention group participants received training in form of audio, image, or video messages with focus on increasing medication adherence via the WhatsApp while the control group received the routine care. Data analysis 16 weeks after initiating the intervention showed no significant difference between the two groups [31]. The results of this study are not consistent with the results of present study and the use of WhatsApp could not lead to a significant difference between two groups. Differences in structures of these apps, including ease of use and observance of delicate considerations required for older adults regarding using the app, which were carefully implemented in designing the app in the present study might be the reason of this discrepancy. Chandler et al. investigated the impact of mobile phone use on treatment adherence in hypertensive patients in the United States. Three months after using the app, treatment adherence improved markedly in the intervention group while no change was observed in control group. Findings showed a significant reduction in systolic and diastolic blood pressure in intervention group compared to control group [32]. The results of this study are consistent with the results of the present study; as mentioned earlier, the designed app reduced blood pressure alterations in the intervention group in the present study.

Finally, Athilingam et al. conducted a study to evaluate the effect of enhanced self-care using mobile technology on reducing readmission in patients with congestive heart failure. Results showed that patients in intervention group were not readmitted during the 30 days of study compared to control group. Preliminary results showed the potential effectiveness of app in reducing readmission and improving self-care in heart failure patients [33], which was similar to the results of the present study. Nonetheless, the sample size was much larger in the present study compared to above study, which improves the generalizability of the results.

## Conclusion

Considering that one of the basic goals of the health care system is to prevent the increase of costs and reduce the economic burden that is created by each individual and group of society, investigations such as the present study will provide valuable information about what policies should be implemented to achieve the stated objective. According to the findings of this study, by improving medication adherence, many adverse events such as re-hospitalization, as the major one, will be reduced, so it could a precious data for policy makers to promote older adults’ quality of life and society welfare by familiarize the elderly with new technologies as much as possible in order to take advantage of it and employ them to manage their health condition effectively. Use of health mobile application designed according to the needs and capabilities of the target group improve the patients’ control over their disease and helps them prevent adverse events.

## Limitations

There were some limitations in this study. Considering Morisky scale as a subjective tool to assess medication adherence, we used pill count method to avoid internal validity threat. This method was used as an objective and supplementary method, considering its practicality and simplicity. Lack of generalizability due to a single site of study was the other limitation. Also lifestyle-related factors with medicine adherence were seldom examined in this study. Larger studies covering wider areas and focusing more on the lifestyle or other risk factors should be carried out in future. Use of a case report form for assessing adverse events in this study was another limitation. Totally self-reported answers may be exaggerated; respondents may be too embarrassed to reveal private details or even forget needed data. Due to neutralize this effect, the investigator compared the answer with their medical records and physician confirmation. The possibility of cross-group contamination also could have affected our study. Although the investigators planned to have even introduction session with each group in different days to avoid participants’ connection.

### Electronic supplementary material

Below is the link to the electronic supplementary material.

Supplementary Material 1

## Data Availability

All data generated in this study are included in the manuscript. Datasets are available upon reasonable request from the corresponding author. Mrs. Pashaeypoor is available for data and materials availability. The available e-mail address is Sh-Pashaeipour@tums.ac.ir.
