# Supplementary material for: Effect of using a mobile drug management application on medication adherence and hospital readmission among elderly patients with polypharmacy: a randomized controlled trial
Source: BMC Health Serv Res. 2023 Nov 2;23:1192. doi: 10.1186/s12913-023-10177-4 (PMC10621100; doi:10.1186/s12913-023-10177-4)
Supplement: Supplementary file 1 — Supplementary Material 1 [file 12913_2023_10177_MOESM1_ESM.docx]

No:

**Case report form of adverse events**

Dear participant

It would be nice of you to answer the first three question of this questionnaire (1, 2, 3) the rest will be completed by your health care providers. Please clarify if you have had experienced any of these items during eight past weeks. Be sure your information will remain confidential. Feel free to ask question if any.

| 1) | Falling | Yes  No |
| --- | --- | --- |
| 2) | Hypo or hypertension | Yes  No |
| 3) | Hypo or hyperglycemia | Yes  No |
| 4) | Re-hospitalization due to disease aggravation | Yes  No |
| 5) | Re-hospitalization due to error in medication consumption | Yes  No |
| 6) | Drug use accuracy (Based of pill count method) | Yes  No |

Any comment:
